# Multisite silicon neural probes with integrated silicon nitride waveguides and gratings for optogenetic applications

**DOI:** 10.1038/srep22693

**Published:** 2016-03-04

**Authors:** Euijae Shim, Yu Chen, Sotiris Masmanidis, Mo Li

**Affiliations:** 1 Department of Electrical and Computer Engineering, University of Minnesota, Minneapolis, MN 55455, USA; 2 Department of Neurobiology, University of California, Los Angeles, CA 90095, USA

## Abstract

Optimal optogenetic perturbation of brain circuit activity often requires light delivery in a precise spatial pattern that cannot be achieved with conventional optical fibers. We demonstrate an implantable silicon-based probe with a compact light delivery system, consisting of silicon nitride waveguides and grating couplers for out-of-plane light emission with high spatial resolution. 473 nm light is coupled into and guided in cm-long waveguide and emitted at the output grating coupler. Using the direct cut-back and out-scattering measurement techniques, the propagation optical loss of the waveguide is measured to be below 3 dB/cm. The grating couplers provide collimated light emission with sufficient irradiance for neural stimulation. Finally, a probe with multisite light delivery with three output grating emitters from a single laser input is demonstrated.

The emergence of optogenetic technologies in neuroscience has provided a plethora of new opportunities for studying the contribution of genetically and anatomically defined neuronal subpopulations to behavior and brain circuit activity^1^,^2^,^3^. Increasingly, optogenetics techniques are being targeted toward relatively small populations of cells in order to isolate their role in neural microcircuit processing during behavior^3^,^4^,^5^,^6^,^7^,^8^.

Glass optical fibers are widely used to deliver light into the brain for optogenetic experiments involving behavioral^9^ and electrophysiological measurements^10^,^11^. Light emission from optical fibers, however, illuminates a large volume of brain and, therefore, lacks the spatial selectivity needed to perturb only small (tens to hundreds) populations of cells. To achieve multisite optical activation or inhibition, implantation of multiple fibers would be required, with which only distant brain regions can be independently controlled due to the large volume illuminated by each fiber. To overcome these challenges, recently a micro-fabricated optical probe with multiple integrated waveguides made of silicon oxynitride (SiON) has been demonstrated for both 473 nm and 632 nm wavelengths^12^. In such a multi-waveguide probe, light is guided with low loss and emits toward the sides of the probe after being reflected by 90° metal reflectors. A large 3D array of such probes has also been developed^13^. However, there remain significant opportunities for using integrated photonics, nanoelectronics and nanofabrication technologies to improve localized, multisite light delivery techniques on implantable devices integrated with large-scale electrophysiological sensing capabilities^14^.

Here we report a microfabricated silicon-based probe with high-index silicon nitride (SiN) waveguides and grating couplers as a new nanophotonic approach of light delivery for optogenetic experiments. The small footprint of these nanophotonic components on a narrow, minimally invasive probe has the potential to provide compact integration with high-density electrode arrays^15^. Figure 1a illustrates a possible configuration of such a multipurpose probe. In contrast to side-emission via reflective surfaces, the new probe uses grating couplers to emit light out-of-plane of the probe, allowing optogenetic stimulation of neurons just above the couplers. This light delivery zone would thus coincide with the region closest to the recording electrodes located on the front surface of the probe. Such a probe could therefore provide simultaneous optogenetic stimulation and electrophysiological recording with high spatiotemporal resolution in small, spatially confined populations of neurons.

## Methods

The fabrication process starts with a silicon on insulator (SOI) wafer with a 1.2 μm oxide layer and a 20 μm Si layer. Thermal growth of a 1 μm oxide is followed by deposition of a 200 nm thick layer of silicon nitride using low-pressure chemical vapor deposition (LPCVD). The stoichiometry of Si_3_N_4_ is very important to lower the absorption of blue light at 473 nm^16^. In contrast, silicon rich nitride, although it affords higher refractive index, can have high absorption at 473 nm. The waveguides and grating couplers are patterned with e-beam lithography and reactive ion etching (RIE) based on fluorine chemistry. After cleaning the devices using Piranha etchant, plasma-enhanced chemical vapor deposition (PECVD) is used to deposit a 1 μm thick oxide layer for top cladding. Once the probe is defined by lithographycally patterned photoresist, the Bosch and other fluorine-based RIE processes are used to etch Si and the rest layers (i.e. oxide and nitride layers), respectively. After protecting the front of the wafer with etch-resistant polymer coating (ProTEK SR), the 400 μm thick Si substrate is etched using the Bosch process. Finally, probes with photonic structures are released when the polymer is removed by organic solvents.

**Figure 1:**
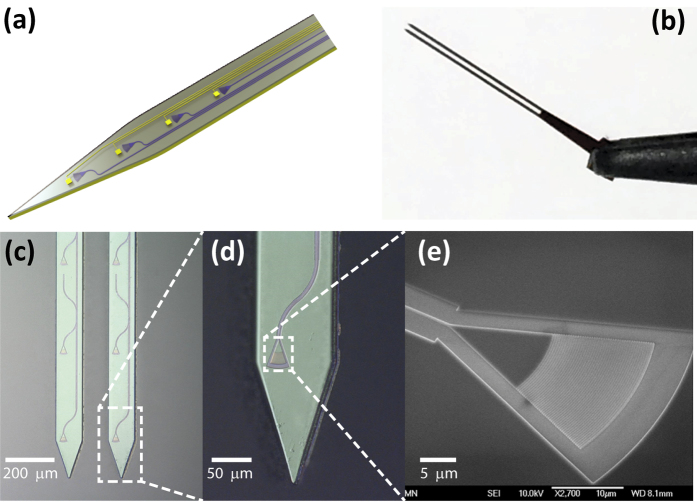
(**a**) Schematic of a hypothetical neural probe with an array of optical waveguides, grating couplers and microelectrodes for high spatiotemporal optogenetic stimulation and electrophysiological recording. (**b**) Photo of a completed double-shank probe held by a tweezer. (**c**) Microscope image of output grating couplers and a waveguide on a double-shank probe. (**d**) Microscope image of a probe tip with a grating coupler connected to a waveguide. (**e**) SEM image of a grating coupler with a length of 15 μm and width of 20 μm.

Figure 1b shows a finished probe with two shanks. Each probe includes four identical grating couplers, one for light input and three for output, and a shallow-etched rib waveguide connecting them. Microscope images of probe tips and a scanning electron microscope (SEM) image of a grating coupler are shown in Fig. 1b–d, respectively. The optimized design of a grating coupler has a size of 15 μm × 20 μm with a grating duty cycle of 0.84 and a period of 350 nm for optimal coupling efficiency at 473 nm. For other wavelengths, the grating parameters can be varied accordingly. The waveguide width starts from 400 nm at the input coupler and adiabatically widens to 2 μm to reduce propagation loss, and tapers back to 400 nm at the output emitter. For comparison, the SiON waveguide used in (ref. 12) is 20 μm wide. Each probe consists of two shanks and each shank is 0.7 cm long and 90 μm wide.

## Results and Discussion

### Experimental Results

To test the waveguide’s optical transmission properties, light from a 473 nm CW diode laser (Optoengine LLC) was collimated into a 3 μm-core single mode fiber. The fiber was aligned to an input grating coupler to couple light into the waveguide. For the probes to be implanted in animals, the fiber arrays will be directly bonded with the grating couplers using the in-line coupling element (ICE) method (PLC Connections LLC). The average coupling efficiency between the fiber and the input grating coupler was determined to be in the range of 10–15% (loss of 8.2–10 dB). The variation in the coupling efficiency is attributed to the variation of the grating fabrication limited by e-beam lithography and etching resolution. After propagation in the waveguide, light emits out-of-plane through the output grating coupler. By using shallow etched gratings with an overlay layer to break the symmetry or buried reflector layers, the output coupling efficiency can be further improved to much less than 1 dB^17^,^18^.

**Figure 2:**
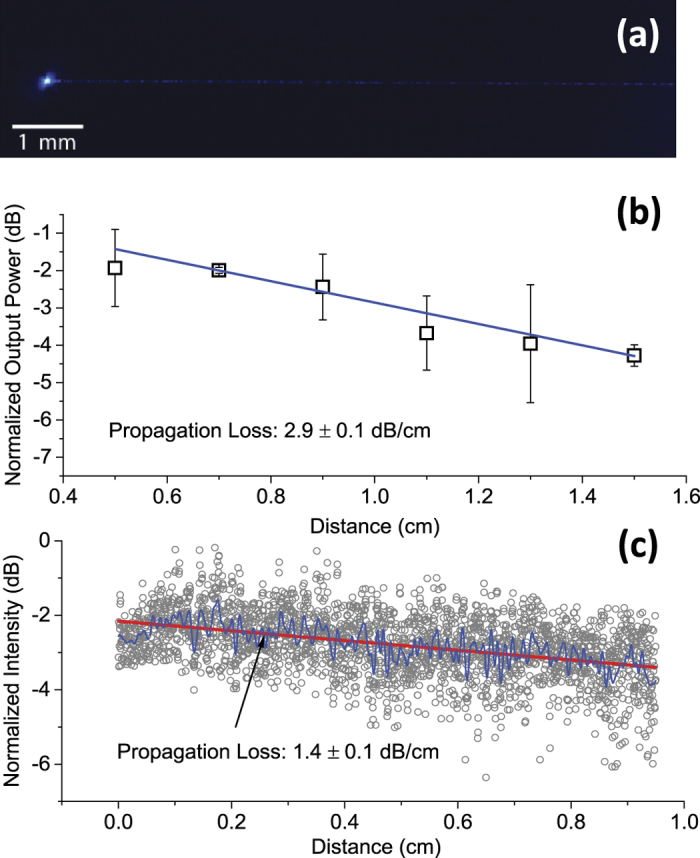
(**a**) Dark-field microscope image of an output grating coupler emitting blue light after transmission through a 1.3 cm long waveguide. (**b**) Direct cut-back measurement for a set of devices. Each set includes devices with 0.5 cm, 0.7 cm, 0.9 cm, 1.1 cm, 1.3 cm, and 1.5 cm long waveguides. Blue line is linear fitting of the data, yield propagation loss of 2.9 dB/cm. (**c**) Out-scattering measurement of a 0.95 cm long, 2 μm wide waveguide. Blue line is low-pass filtering of the data (grey symbols) obtained from imaging processing of (**a**). Red line is the linear fitting, yielding a propagation loss of 1.4 dB/cm.

Two measurement methods were employed to evaluate the propagation losses of the waveguides. One was the direct cut-back method and the other was by imaging out-scattered light from the waveguide. Each device used for measurements has two grating couplers for input light and output, and a waveguide connecting them. For the direct cut-back method, a set of waveguides with five different lengths (0.5 cm, 0.7 cm, 0.9 cm, 1.1 cm, 1.3 cm, and 1.5 cm), each with identical grating couplers and fabricated on the same substrates, was measured. Figure 2b shows the results of output power plotted versus waveguide length. Linear fitting of the data gives the propagation loss of 2.9 ± 0.1 dB/cm, which is comparable to the value of 3.0 dB/cm obtained in the much larger SON waveguides. A rib waveguide design helped achieve the sub-3 dB/cm propagation loss by spreading mode intensity distribution and reducing the effective sidewall roughness compared to a ridge waveguide. Because the loss is mainly attributed to scattering and distributed evenly along the waveguide, heating effect of the waveguide and the surrounding tissue is trivial. We believe the loss can be further reduced by improving the lithography resolution and the etching recipe.

The out-scattering method is based on the assumption that the optical intensity in the waveguide is proportional to the brightness of light scattering out of the waveguide due to uniform distribution of surface roughness. After light was coupled to a waveguide, images of different regions of the waveguide were taken with a CCD camera. Since this measurement is based on relative brightness in the image, it is important that each pixel used for image processing should not be saturated and all images should be captured at the same exposure and illumination conditions. An example of such an image is shown in Fig. 2a. Following image processing by MATLAB, the brightness of each section of a waveguide can be plotted over the waveguide length to calculate the propagation loss. Figure 2c shows the out-scattering measurement of a 0.95 cm long waveguide taken from a 1.3 cm long device. The propagation loss is determined to be 1.4 ± 0.1 dB/cm. The 1.5 dB/cm of propagation loss difference between two methods is mainly attributed to variance in grating coupler fabrication, due to the lithography resolution limit and fabrication imperfections.

### Simulation Results

**Figure 3:**
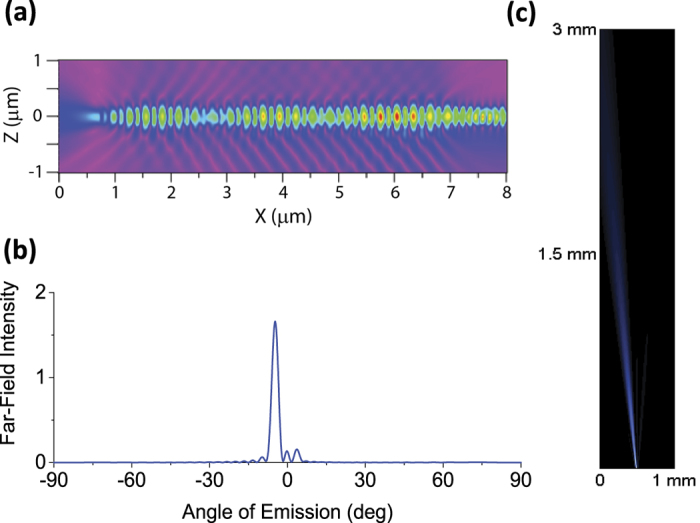
Simulated intensity profile in scattering medium. (**a**) Near-field electric field profile of a waveguide and a grating coupler in the cross-sectional view. (**b**) Far-field intensity of the emitter calculated from the near-field data. (**c**) 2D color map of emitted output light intensity from a grating coupler.

To further characterize the expected device performance when implanted in brain tissue, we simulated the optimized design of our waveguide and grating coupler in strongly scattering medium using FDTD simulation. Light is guided in the waveguide and emits at the grating coupler in both upward and downward directions with a calculated near-field pattern as shown in Fig. 3a. This agrees well with the 3 dB loss of the output grating coupler measured above. Figure 3b shows the far-field angular intensity profile of the light emission, numerically calculated from the near-field data in Fig. 3a. The result shows that the grating coupler emission is highly collimated with a far-field half-angle divergence of 1.5°. The calculated beam parameter product (BPP) is 1.7 × 10^−6^ m·rad, significantly smaller than that of other integrated schemes of emitters, such as SU-8 and oxynitride waveguides^12^,^19^ and μLED^20^. Using this profile and the Kubelka-Munk model of light scattering in biological tissue, the 2D map of emitted light scattering in mouse brain (refractive index: *n* = 1.36; scattering coefficient: *S* = 11.2 mm^−1^ ref. 21) is calculated and plotted as shown in Fig. 3c. Note the beam emitted from the output coupler is at an 8° angle from the normal direction and this angle can be changed through grating design. Taking into account all of the optical losses in our measurement system, including fiber-to-waveguide loss of 8.2–10 dB, waveguide propagation loss of 2.9 dB/cm, and output coupler loss of 3 dB, the optical power emitted at the output coupler is ~190 μW for a 1 cm long device when the optical power at the input fiber is 7 mW. The corresponding irradiance at the plane just above the output coupler is ~630 mW/mm^2^. Assuming the activation threshold of channelrhodopsion-2 labelled neurons is 1 mW/mm^2^ (ref. 21), the Kubelka-Munk model predicts that neurons as far as 1.71 mm away from the output coupler can be activated at maximum laser power. However, since our goal is to modulate only small neural populations in close proximity to the probe, the device could be operated at a fraction of maximal power or multiple waveguides could be operated in parallel. In future study, we will validate above results in phantom brain tissue.

### Multisite Light Delivery

**Figure 4:**
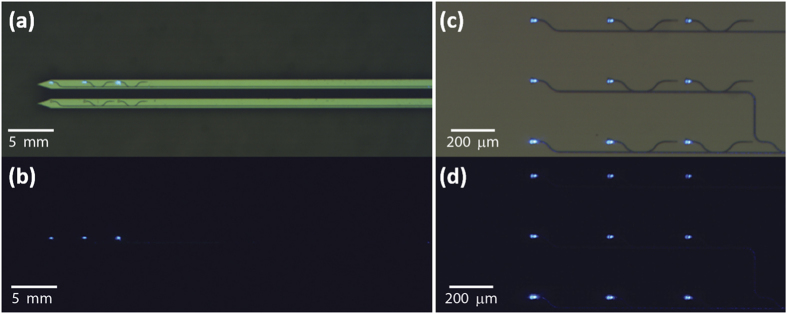
Bright (**a**) and dark (**b**) field optical images of a 1 × 3 array of grating couplers on probe tips emitting 473 nm light from a single laser input. Bright (**c**) and dark (**d**) field optical images of 9 output grating couplers in a 3 × 3 array on chip emitting 473 nm light from a single laser input.

Finally, to achieve multisite light delivery we demonstrate a 1 × 3 array on a probe connected to a single waveguide input and 9 grating emitters in a 3 × 3 array (area: 720 μm × 560 μm) on chip, as shown in Fig. 4a,c, respectively. Directional couplers with different power splitting ratios are designed to approximately evenly distribute input light to each of the grating emitters. The dark field images in Fig. 4b,d show the emission from the output couplers with small intensity variations due to fabrication imperfection. Such an integrated photonics multiplexing approach potentially can allow multisite optical stimulation using only one laser input source. Furthermore, although challenging, it is possible to engineer on-chip switches and modulators to achieve spatially and temporally patterned optical stimulation^22^.

## Conclusion

In conclusion, we have demonstrated an implantable, minimally invasive silicon-based neural probe, integrated with compact silicon nitride waveguides and grating couplers for multisite light delivery. For 473 nm light, the propagation loss in the waveguide is determined to be below 3 dB/cm. Although the current measurement system has relatively high optical insertion loss, in principle the devices are capable of providing sufficient irradiance to activate neurons within the detection range of co-fabricated microelectrodes. For portable use, the grating coupler can be directly bonded on the probe using the in-line coupling element (ICE) configuration (PLC Connections). Future embodiments of such integrated light delivery and electrophysiological recording systems can advance our understanding of brain circuit function through spatially selective multisite optogenetic manipulations.

## Additional Information

**How to cite this article**: Shim, E. *et al*. Multisite silicon neural probes with integrated silicon nitride waveguides and gratings for optogenetic applications. *Sci. Rep*. **6**, 22693; doi: 10.1038/srep22693 (2016).
